# HIV-positive clients of female sex workers in Hunan Province, China: a mixed methods study assessing sexual relationships and risk behavior by type of partner

**DOI:** 10.1186/s12889-019-7446-1

**Published:** 2019-08-16

**Authors:** Peili Wu, Willa M. Dong, Keming Rou, Wei Dong, Chu Zhou, Xi Chen, Jun Zheng, Sarah R. Scott, Zunyou Wu

**Affiliations:** 1 School of Public Health, Anhui Medical University, Hefei, Anhui China; 2 National Center for AIDS/STD Control and Prevention, Chinese Centers for Disease Control and Prevention, 155 Changbai Road, Changping District, Beijing, 102206 People’s Republic of China; 3 Hunan Provincial Center for Disease Control & Prevention, Changsha, Hunan China

**Keywords:** HIV infection, Clients of female sex workers, Prevalence, Risk sexual behaviors, China

## Abstract

**Background:**

In China, clients of female sex workers (CFSWs) have a low rate of condom use and a high prevalence of human immunodeficiency virus (HIV). However, little is known about the high-risk sexual behaviors of HIV-positive CFSWs.

**Methods:**

In 2014, 327 CFSWs diagnosed with HIV for 6 months or longer completed a face-to-face questionnaire for a quantitative survey. In addition, 32 HIV-positive CFSWs were recruited to participate in in-depth interviews (18 participated in both, 14 participated in-depth interviews only) to explore reasons for extramarital sexual behaviors and inconsistent condom use. The quantitative data on sexual risk behaviors were analyzed using chi-square tests. Interviews were coded inductively for emerging themes.

**Results:**

Among the participants of the quantitative survey, 41.6% (136/327) had sex with regular sexual partners only in the past 6 months, of whom 64.0% (87/136) had consistent condom use; 27.5% (90/327) of the participants had sex with irregular sexual partners in the past 6 months, of which, 46.7% (42/90) had consistent condom use. The qualitative study suggested that HIV positive sero-status, willingness to protect their spouses or regular sexual partners, and lacking a sense of responsibility to protect their commercial and casual sexual partners, influence CFSWs’ sexual behaviors.

**Conclusions:**

HIV-positive CFSWs continue to practice unsafe sexual behaviors with regular and irregular partners after HIV diagnosis, but were more willing to protect their regular partners. Future interventions targeting HIV-positive CFSWs should not only be confined to sero-discordant couples, but also need to instill a sense of responsibility to protect the commercial and casual partners and reduce the number of concurrent partners.

## Background

In China, the sexual transmission of HIV is rapidly increasing [1, 2]. Heterosexual transmission accounted for 52.2% of the estimated new infections in 2011, compared to 11.3% prior to 2006 [3]. For people living with HIV (PLHIV), improvements in health and quality of life due to anti-retroviral therapy (ART) have enabled many to engage in sexual activity [4, 5]. In China, the few interventions for PLHIV mainly focus on HIV discordant couples [6, 7]. More so, behavioral interventions typically aim to prevent new infections among key affected populations, rather than focusing broadly on all PLHIV [1]. Owing to rapid changes in sexual behaviors and attitudes over the past three decades, extramarital sex and concurrent sexual relationships are increasingly common in China [8]. Therefore, current interventions may not adequately address the range of sexual relationships PLHIV may have.

In 2006, 4.2% of Chinese men were estimated to have paid for sex in the past year [9]. The large numbers of clients of female sex workers (CFSWs) that fuel the flourishing sex industry are likely to play an important role in heterosexual transmission of HIV. CFSWs have a high HIV and sexually transmitted infection (STI) prevalence, low consistent condom use rate, high rates of partner exchange or concurrent sexual partners, and decisive power on safe sex [10–13]. For example, a study of 600 clients in Sichuan found that the syphilis prevalence was 8.7%, and consistent condom use with FSWs was 30.5% [10]. Additionally, CFSWs may be very sexually active as over one-third have casual sexual partners and 39% reported purchasing sex more than once a month [12]. However, most preventive interventions targeting commercial sex focus on FSWs [11] while the majority of research on CFSWs only reports HIV/STI prevalence or condom use rate for the general client population [10, 12].

After being diagnosed with HIV, interventions shift to preventing secondary transmission. Despite the need to understand the behaviors and relationships of HIV-positive CFSWs that shape secondary transmission [14], there are no studies specifically focusing on this group in China. In this study, we used mixed methods to examine current sex behaviors, relationships, types of partners, and unprotected sex among different sexual partners of HIV positive CFSWs, in order to understand the reasons for unsafe sex behavior.

## Methods

### Mixed-methods study design

This study used a convergent, parallel, mixed-methods design to develop an understanding of HIV positive CFSW’s sexual risk behavior [15]. Current sexual risk behavior and associated factors were first collected by a questionnaire. The context of sexual relationships and condom use were explored through semi-structured, in-depth interviews. After analyzing the quantitative and qualitative data separately, we merged the results of the two strands to describe the sexual risk behavior of HIV positive CFSWs.

### Study sites

Changsha and Hengyang are adjacent, and are the capital city and the second largest city in Hunan Province in central China. The sex industry in both cities includes a variety of sexual service venues; including nightclubs, karaoke parlors, hotels, bathhouses, hair salons, massage parlors, and roadside restaurants. Changsha and Hengyang were chosen as the study sites because they have the most PLHIV in Hunan, and heterosexual sex is the main transmission route of HIV. By the end of 2013, there were 1347 PLHIV in Changsha, of which 963 were receiving ART in Changsha, and 1051 PLHIV in Hengyang, of which 689 were receiving ART.

### Participants

The participants of the quantitative study were conveniently recruited from ART clinics and local Centers for Disease Control (CDCs) in Changsha and Hengyang between March and June 2014. First, all HIV positive heterosexual men were identified by outpatient doctors at an ART outpatient clinic, or through reviewing clinic medical records which record the route of HIV transmission. Second, any of the above men who reported having unprotected sex with a FSW before his HIV diagnosis and also denied the possibility that his regular partner transmitted HIV to him, were included. Third, HIV positive heterosexual men who also reported having no history of injection drug use or having anal or oral sex with men before their HIV diagnosis were considered. PLHIV who met the above three criteria were considered HIV-positive CFSWs. Study inclusion criteria included: (1) meeting all of the criteria of HIV positive CFSWs; (2) being 18 years of age or older; (3) being aware of their HIV positive sero-status for at least 6 months; (4) being able to provide written, informed consent.

For the qualitative study, participants (*n* = 32) were recruited after completing the questionnaire or were referred by health care workers and community based organizations serving PLHIV. In addition to meeting the above inclusion criteria, the participants were first sampled to maximize differences in age, occupation, education, and sexual behaviors after HIV diagnosis. Participants were then sampled purposively according to the purpose of the study.

### Data collection

#### Quantitative data collection

After providing informed consent, participants answered questions on demographic characteristics, HIV related knowledge, ART usage, and sexual behaviors with different sex partners (regular sexual partners, commercial sexual partners, and casual sexual partners) in the past 6 months. Trained study staff administered a structured questionnaire during face-to-face interviews in a private room in ART clinics or local Centers for Disease Controls (CDCs) study sites. Study staff were nurses working at the ART clinics or epidemiologists from the local CDC sites, both of whom have regular contact with PLHIV. Staff were trained on the purpose of the study, informed consent, and the requirements for conducting the study.

Regular sexual partners were defined as a spouse or long-term sexual partner with whom the participant had regular sex without monetary compensation. Commercial sexual partners referred to female sex workers (FSWs) to whom the participant was required to pay money to have sex, and a casual partner was someone with whom the participant had sex with occasionally without compensation. Being sexually active was defined as having sex in the past 6 months and consistent condom use was defined as using condoms every time during sex in the past 6 months.

#### Qualitative data collection

In the qualitative study, 32 participants were interviewed in private rooms at ART outpatient clinics in Hengyang. These interviews explored attitudes and reasons for extramarital sex and unsafe sex behaviors amongst PLHIV. A total of 32 participants took part in semi-structured, in-depth interviews and 18 of these participants had also completed the questionnaire during the quantitative survey. Each interview lasted 30–90 min and was conducted in Mandarin by the first author, a female native Chinese speaker (WPL). All interviews were audio recorded. Interviews were conducted until data saturation was reached. Interview excerpts were translated separately by the first two authors (WPL and WMD, a native English speaker), and then discussed to choose the best translation, which was verified by another author (ZC) proficient in both English and Chinese.

### Data analysis

#### Quantitative analysis

All quantitative data were entered with EPIDATA 3.1 (EpiData Association, Denmark) and analyzed with SPSS16.0 (SPSS Inc., Illinois, USA). Descriptive demographic variables, ART status, and risky sexual behavior were presented as categorical variables. Sexual behavior among different sex partners was compared with a chi-square test. Statistical significance was set at *P* < 0.05 for all tests.

#### Qualitative analysis

Digitally recorded interviews were transcribed verbatim and checked against the original audio recording for accuracy. Data analysis was conducted simultaneously with data collection. Line-by-line initial coding was performed first on transcribed interviews, and codes were developed based on emergent themes. A codebook was then developed and these codes were applied to the rest of the transcripts. Additionally, theoretical memos were written to explore emergent themes. ATLAS.ti5.0 (Scientific Software, Berlin, Germany) was used to code and manage qualitative data.

### Ethical considerations

This study received approval from the Institutional Review Board of the National Center for AIDS/STD Control & Prevention, China CDC. All participants provided written consent. Participants of the quantitative and qualitative studies were compensated 30 RMB (renminbi, currency of the People’s Republic of China, or approximately $4 United States Dollars USD) and 50 RMB (approximately $7 USD), respectively, for their time and travel costs.

## Results

### Quantitative results

We recruited and interviewed 378 CFSWs. Among them, 327 CFSWs (86.5%, 327/378) finished the survey, met the questionnaire quality requirement, and were included in the quantitative study. Of 327 CFSWs, 97 were recruited in Changsha, with 80 participants from the ART clinics and 17 via local CDC study sites, while 230 were recruited from Hengyang, with 165 participants from the ART clinics and 65 via the local CDC study sites.

#### Demographics and clinical characteristics

The mean age of the sample was 46.4 years. More than one-fifth of the participants had not finished nine years of compulsory education. Around one-third were migrant laborers (103/327) and 25.7% (84/327) were formally employed in enterprises. Over half of the men (191/327) were married and 27.8% (91/327) had migrated for work in the past 6 months. In addition, over four-fifths of the participants were receiving ART (Table 1).

**Table 1:** Demographic and HIV^a^-related characteristics of HIV positive clients of females sex workers in Changsha and Hengyang in 2014

Variables	Quantitative (***N*** = 327)	Qualitative (N = 32)
	n (%)	n (%)
Study sites		
Changsha	97 (29.7%)	8 (25.0%)
Hengyang	230 (70.3%)	24 (75.0%)
Age (years)		
≥ 50	125 (38.2%)	14 (43.8%)
< 50	202 (61.8%)	18 (56.2%)
Education		
< 9 years of school	186 (56.8%)	21 (65.6%)
≥ 9 years of school	141 (43.1%)	11 (34.4%)
Marital status		
Divorced/separated/Windowed/never married	136 (41.6%)	9 (28.1%)
Married/remarried	191 (58.4%)	23 (71.9%)
Occupations		
Farmer	68 (20.8%)	11 (34.4%)
Migrant laborer	103 (31.4%)	8 (25.0%)
Enterprises staff	84 (25.7%)	7 (21.9%)
Business	41 (12.5%)	6 (18.8%)
Others	31 (9.5%)	–
Monthly income (RMB^b^)		
0–999	92 (28.1%)	10 (31.3%)
1000-2000	92 (28.1%)	7 (21.9%)
2000-3000	87 (27.1%)	4 (12.5%)
> 3000	56 (17.1%)	11 (34.4%)
Last CD4 count		
< 350	197 (60.2%)	27 (84.4%)
≥ 350	104 (31.8%)	5 (15.6%)
Unknown	26 (8.0%)	–
Currently on ART^c^	275 (84.1%)	27 (84.4%)
Migrated for work in last 6 months	91 (27.8%)	25 (78.1%)
HIV knowledgeable	273 (83.5%)	–
Knew PLHIV^d^ on ART may infect sexual partners	212 (64.8%)	–
Heard of HIV before diagnosis	81 (24.8%)	4 (12.5%)

^a^*HIV* human immunodeficiency virus, ^b^*RMB* renminbi, the currency of the People’s Republic of China, ^c^*ART* anti-retroviral therapy, ^d^*PLHIV* people living with HIV

#### HIV related knowledge

Of the 327 participants, 83.5% (273/327) were HIV knowledgeable, defined as answering six or more HIV-related questions correctly out of a total ten questions. Only 64.8% (212/327) knew that PLHIV receiving ART may still infect their sexual partners. One-quarter of HIV-positive clients (24.8%, 81/327) had never heard of HIV until they were diagnosed.

#### Sexual behaviors, partner types and condom use

The mean age for first sexual behavior was 22.6 years. Among the 280 married or ever married participants, 44.6% (125/280) had premarital sex. The mean age for first purchase of commercial sex was 31.4 years, with a range from 16 years to 70 years. Before their HIV diagnosis, 52.0% (170/327) had ever used condoms and 27.2% (89/327) had a history of sexually transmitted infections. In the past 6 months, 20.8% (68/327) had sex with commercial sexual partners and 9.2% (30/327) had sex with casual partners. Consistent condom use over the past six months was 47.1% (32/68) with commercial sexual partners and 43.3% (13/30) with casual partners (Table 2).

**Table 2:** Sexual behaviors and HIV^a^ related knowledge in the past six months of HIV-positive clients of female sex workers in Changsha and Hengyang in 2014

Sexual behaviors and relationships	%	*N* = 327 (n/N)
Had a regular sexual partner	65.4	(214/327)
Had sex (past 6 months)	63.6	(136/214)
Consistent condom use (past 6 months)	64.0	(87/136)
Condom use at last sex	74.3	(101/136)
Had sex with commercial sexual partner	20.8	(68/327)
Consistent condom use (past 6 months)	47.1	(32/68)
Condom use at last sex (past 6 months)	64.7	(44/68)
Had sex with casual sexual partner	9.2	(30/327)
Consistent condom use (past 6 months)	43.3	(13/30)
Condom use at last sex (past 6 months)	60.0	(18/30)
Had ever used condoms before HIV diagnosis	52.0	(170/327)
Heard of HIV/AIDS^b^ before HIV diagnosis	75.2	(246/327)
Ever had STD^c^ before HIV diagnosis	27.2	(89/327)

^a^*HIV* human immunodeficiency virus, ^b^*AIDS* acquired immune deficiency virus, ^c^*STD* sexually transmitted disease

With regular partners, consistent condom use in the past 6 months was 2.0 times higher than with commercial partners (*P* = 0.021, χ^2^ = 5.334) and 2.3 times higher than with casual partners (*P* = 0.037, χ^2^ = 4.370). There was no difference in consistent condom use between commercial partners and casual partners (*P* = 0.733, χ^2^ = 0.116) (Table 3).

**Table 3:** Condom use in the past six months and at last sex among different types of relationships of clients of females sex workers in Changsha and Hengyang in 2014

Relationship type	Consistent condom use in the past six months		Condom use at last sex	
	*p-value*	χ^2^	*p-value*	χ^2^
Regular vs. Commercial	0.021*	5.344	0.156	2.015
Regular vs. Casual	0.037*	4.370	0.116	2.464
Commercial vs. Casual	0.733	0.116	0.656	0.198

*p value significant at < 0.05

### Qualitative results

#### Participants

The age of the 32 HIV positive CFSWs ranged from 25 to 67 years. The majority were married (23/32) and had less than nine years of education (21/32). Participants reported a monthly income between 0 to over 3000 RMB, with a median monthly income of 2200 RMB (roughly $320 USD), which is the average income of most middle-income positions in China. In the past 6 months, eight participants had sex with FSWs or casual partners, nine had sex with wives, and one had concurrent partners (Table 1). Several general themes were identified through the qualitative assessment, including participants’ feelings and beliefs towards extramarital sexual behavior; sexual relationships with different types of partners; and condom use during sexual relations with community sex workers. Table 4 displays these findings in relation to condom use. Quotes were chosen to be representative of the emerging themes and provide authenticity to the results.

**Table 4:** Factors influencing consistent condom use by partner type based on findings from a qualitative survey amongst 32 HIV positive clients of females sex workers in Changsha and Hengyang in 2014

Regular Partners		Casual/commercial partners	
Promote	Discourage	Promote	Discourage
✓ Negative HIV^a^ status of sexual partners; ✓ HIV disclosure; ✓ Good relationships; co-infection knowledge; ✓ Feelings of responsibility; ✓ Have sex with regular sexual partners; ✓ Good conscientiousness and quality; ✓ Sex behavior counseling and education	✓ HIV non-disclosure; ✓ Bad relationships; ✓ Condom reduce sex pleasure; ✓ Alcohol use; ✓ HIV stigma; ✓ Lack of consciousness to protect others	✓ Co-infection knowledge; ✓ Good conscientiousness and quality; ✓ Sex counseling and education;	✓ Alcohol use; ✓ Condom reduce sex pleasure; ✓ Lack of consciousness to protect others ✓ Familiar with the sexual partners; ✓ HIV positive status of sexual partners; ✓ Discrimination of FSWs^b^

^a^*HIV* human immunodeficiency virus, ^b^*FSWs* female sex workers

#### Attitudes towards extramarital sexual behavior

Almost all participants reported that extramarital sexual behavior, including sex with both commercial and noncommercial partners, was popular among their social networks. Participants frequently referred to extramarital sex as “going out to play” (*qu waimian shua*), that it was an act of fun without responsibility, that others in their circle were also participating in. One construction businessman who lived in nearby Shaoyang city but received ART in Hengyang reported: “Almost 50% of men in Shaoyang have gone to massage parlors or other sex service venues. In my circle of friends [in architecture and real estate], it’s 100%. It’s a trend, and sometimes I also invite them to banquets” (code No. 006, 47 years). Extramarital sexual behavior was perceived positively among peers, serving as a means of conversation and gloating. One tour bus driver explained, “Friends like to talk about those things [meaning casual sex], when they get together. It seems extraordinary if he had sex with several women, and is a thing worth boasting about” (code No. 014, 41 years).

Participants reported various motivations for having extramarital sex; including meeting their physiological needs; to add excitement to a monotonous life; peer pressure; or as part of banquets where business partners build relationships through dining, drinking, and engaging in sex with FSWs. Some participants also reported extramarital sex behavior signaled men’s social status and wealth, as it reflected that he had enough money to afford the expenses of his mistress. “As the saying goes, ‘men become bad as soon as they have money’. It’s true, if I didn’t have money, I wouldn’t have spare money to go out to play, it’s related”, a businessman reported (code No.012, 48 years).

Extramarital relationships were seen as acceptable because they were not seen as affecting a man’s ability to fulfill his family obligations, which were the most important. A taxi driver stated: “[extramarital sex] is very normal. I don’t think it matters, after all, it is loveless and it’s separate from your family responsibility” (code No.002, 40 years). A business man who had a casual partner also reported: “I am a man responsible for my family, my wife didn’t abandon me when I was sick, so when I go out to play, if something happens in my family, I must go home if my family gives me a call. Nothing is more important than family” (code No.005, 34 years).

#### Sexual relationships with different types of partners

Though participants knew using condoms could prevent HIV transmission, many CFSWs reported that after becoming diagnosed with HIV, they stopped having sex with their wives as they saw abstaining from sex with their wives as essential to prevent infection. One participant stated that “my wife is negative, I am afraid of infecting her, this disease is too terrible, I can’t transmit it to her” (code No.008, 58 years). Participants reported a lack of partners and an inability or unwillingness to have sex with their regular partners as important reasons for having sex with FSWs. “Now, I can’t sleep with my wife, she doesn’t have the disease, she is afraid and she does not dare to be with me. When I want [to have sex], I can only spend some money and go out [to find FSWs]” (code No.001, 47 years). In addition, participants viewed FSWs as viable sexual partners because of their HIV infection: “This disease has produced a huge shade in my heart. To put it simply, if I didn’t have this disease, I will look for a normal girl, but the fact is that I’m HIV positive. How can I let off [my libido]? I go out and look for one [FSW]” (code No.007, 33 years).

Two participants reported having casual, non-commercial partners. An unmarried young man who had broken up with his girlfriend before his HIV diagnosis did not feel ready to date after becoming infected. He felt hopeless, as he thought no girls would be willing to be his girlfriend or marry him if they knew he was infected with HIV. In order to fulfill his sexual needs, he found one-night lovers on a popular instant messaging app. A married man found an HIV-positive partner with whom he could share feelings, as he felt that his status produced a barrier to intimacy with his HIV-negative wife. He initiated a relationship with a woman he had seen at an ART clinic because he wanted a partner who also had the disease to communicate with.

#### Condom use in HIV positive CFSWs’ sexual relationships

Table 4 presents models for consistent condom use with different types of partners.

After being diagnosed, married participants used condoms consistently with their HIV negative spouses. “I try my best not to have sex with my wife, she doesn’t have this disease, if we have sex, we use condoms 100 percent. She didn’t abandon me when I was totally helpless, so I can’t betray her” (code No. 005, aged 34). The only participant who did not use condoms with his wife concealed his status from her, and did not know her HIV status. He explained after his first wife died, he remarried but he and his second wife did not have a strong emotional attachment.

With FSWs, participants reported they used condoms consistently to avoid infecting others. The one participant who never used condoms reported that he usually went to bath houses or massage parlors one to two times monthly, which had been greatly reduced compared to before his HIV diagnosis. He explained that he did not use condoms because “FSWs don’t ask me to use condoms, and I feel uncomfortable when using condoms. I didn’t like it” (code No. 022, 54 years). One participant who consistently used condoms described the motivations of PLHIV who engage in unsafe sexual behavior: “Human beings are all selfish, FSWs are not family. For me, I have a strong desire to protect my family, but when I go out to play, I don’t think too much” (code No. 007, 33 years).

Alcohol also hindered participants’ condom use, especially when drinking with friends. As a participant reported, “when you drink, you can make love for a longer time, and if you use condoms it would greatly influence your sexual pleasure” (code No. 002, 40 years). Another participant reported, “you can’t understand, when you drink with your friends, you will be indulging in the pleasure, no one will think of condoms” (code No. 007, 33 years).

Condom use with FSWs was also framed as a matter of conscientiousness (*liangxin*) and overall quality of the individual (*suzhi*). A divorced man reported, “I can’t understand why people don’t use condoms after knowing they are infected with HIV. Because to be human, you must at least follow your conscience” (code No. 014, 41 years). Another participant noted, “a man who has *liangxin* and morality and a man who has *suzhi*, he will restrain his own behavior after becoming infected with HIV, just like me, I am very careful when I am with my ‘friend’” (code No. 020, 37 years).

The results of this qualitative study add context and meaning to the outcomes of the quantitative assessment. The rate of condom use was two times higher among participants engaging in sexual relations with regular partners compared to commercial sex workers. The in-depth interviews conducted in this study help elaborate on potential reasons and understandings for such behaviors.

## Discussion

Our mixed-methods study found that clients continued to practice extramarital sex and low rates of consistent condom use, which placed both their regular and casual sexual partners at risk of infection. Additionally, consistent condom use varied by type of partner. The qualitative results suggest that interventions need to address the emotional and sociocultural factors underpinning these disparities in condom use. To our knowledge, this study is the first to focus on the sexual risk behaviors of HIV-positive CFSWs in China.

CFSWs’ consistent condom use was significantly higher with regular partners (64.0%) than with commercial (47.1%) partners. The quantitative findings confirm clients’ low levels of condom use in commercial sex settings in China [8, 10]. The qualitative data may explain this difference. In the interviews, participants described feeling a strong sense of responsibility to protect their family from the consequences of their infection and the prioritization of family obligations. Across settings, the desire to protect one’s family has been found to be a strong motivating factor for PLHIV to practice safer sex with regular partners, especially for couples with a positive relationship [16, 17]. In China, in particular, the behavior of PLHIV is influenced by considerations for the welfare of the family [18]. In contrast, because commercial or casual sexual partners are outside of the family, clients did not feel a sense of obligation. However, the low rates of condom use in commercial sex settings indicates that a stronger and culturally resonant sense of obligation to protecting other partners needs to be instilled. Factors such as perception of HIV risk, which have been found to promote condom use for the general client population, are irrelevant for clients who already have HIV [19]. As our study found that HIV-positive clients (who are receiving treatment and are already exposed to prevention services) have high levels of HIV knowledge. Thus, interventions could also incorporate sociocultural concepts, such as appealing to clients’ sense of their own “suzhi” (quality of individual) and increasing feelings of “liangxin” (conscientiousness) towards sex workers to promote condom use outside of regular relationships.

In China, interventions for PLHIV focus on preventing secondary transmission within sero-discordant couples [6, 7]. However, about 30 % of HIV-positive CFSWs in this study continued having extramarital sex in the past six months, including commercial sex. Our findings on clients’ motivations for seeking commercial sex are consistent with the results of past studies on CFSWs in China, where reasons included peer pressure, stress reduction, lack of regular sexual partners, alcohol use, building social or business relationships, and fulfilling a need for intimate and emotional support [2, 20–22]. However, the qualitative results highlight the role of an HIV-positive status and stigma against HIV in shaping sexual relationships and behaviors that may differ from the general client population. Experiences of HIV-related stigma may lead to anxiety and unsafe sexual behavior amongst PLHIV [23, 24]. As described in the interviews, a positive HIV status motivated clients to seek extramarital and commercial sex to cope with sexual rejection from regular partners; find someone who could understand their experiences; and protect their families from HIV by meeting their sexual needs through others.

HIV-positive clients’ reasons for seeking commercial sex are also rooted in stigma against sex workers. Sex workers were seen as viable partners because clients did not consider them to be “normal” women who would reject them because of their HIV status. Combined with many clients’ lack of condom use in commercial sex settings and social activities, sex workers are at risk for infection given the potentially large numbers of men who continue to seek commercial sex after infection. Current interventions for PLHIV must address prevention in the context of extramarital relationships. While the qualitative findings suggest that HIV-positive clients do not want to infect sex workers, interventions must bridge the gap between this intention and actual condom use practice. In addition, removing discrimination and stigma associated with HIV and sex work in society may also decrease unsafe sexual behavior.

### Limitations

All findings should be interpreted within the study limitations. Most participants were receiving ART treatment. Because those who receive treatment have had contact with HIV-related services, such as counseling, they are more likely to be knowledgeable about HIV. Our study may have overestimated HIV knowledge among HIV-positive clients as a whole. Since 2004 however, all people living with HIV in China are provided ART therapy at no cost, and since 2016, free ART to all PLHIV regardless of CD4 level is recommended. Thus, the population recruited in this study that received treatment would be representative of the larger HIV infected population in the country. In addition, not only were there were several sensitive questions on sexual behaviors and buying sexual services on the study questionnaire, but when we conducted the study, there was also a campaign against sex work in China [25] and deliberately transmitting HIV to others is illegal [26]. Because of social desirability bias, participants may have underreported stigmatized and/or illegal behaviors such as extramarital sex, casual sex, and buying sex, and over-report the rate of consistent condom use. In the quantitative study, over 40% of participants reported consistent condom use with irregular sexual partners, while very few men admitted to not using condoms with sex workers in the qualitative study, which limited the richness of data on reasons for inconsistent condom use. This bias may be especially pronounced in the qualitative study because all interviews were recorded. Extensive measures were taken to protect participant privacy, to promote a trusting environment, and support anonymity of results. Trained researchers with experience working with this population conducted all data collection, with all personally identifiable information removed in order to reduce such biases. Additionally though, future qualitative studies should aim to include more men who use condoms inconsistently to better understand the context of this behavior. Our sample could have been expanded to other study sites in order to increase the likelihood of identifying such individuals. These studies could also assess the barriers to condom use in order to gain a holistic perspective on participants’ decision-making. Lastly, because of convenience sampling and the limited geographic range, this sample may not be representative of HIV positive CFSWs of China as a whole. However, our study examines the sexual behaviors of a population that has rarely been studied in China.

## Conclusions

HIV-positive CFSWs continue to practice unsafe sexual behaviors with regular and irregular partners after an HIV diagnosis, but were more willing to protect their regular partners. Future interventions targeting HIV-positive CFSWs should not only be confined to sero-discordant couples, but also need to instill a sense of responsibility to protect commercial and casual partners and reduce the number of concurrent partners. The results of this mix-methods study can be used to adapt current HIV prevention messaging to promote the agency of FSWs as partners and the importance of condom use when engaging in casual relationships. More so, efforts to promote FSW’s ability to request and use condoms in such relationships could help reduce the potential transmission of HIV in these groups.

## Data Availability

The datasets used and/or analyzed during the current study are available from the corresponding author on reasonable request.

## Abbreviations

ART: Anti-retroviral Therapy
CDCs: Centers for Disease Control
CFSWs: Clients of Female Sex Workers
FSWs: Female Sex Workers
HIV: Human Immunodeficiency Virus
PLHIV: People Living with HIV
RMB: Renminbi
STI: Sexually Transmitted Infections
USD: United States Dollars
